# Tissue Regeneration: The Dark Side of Opioids

**DOI:** 10.3390/ijms22147336

**Published:** 2021-07-08

**Authors:** Cécile Dromard Berthézène, Lise Rabiller, Géraldine Jourdan, Béatrice Cousin, Luc Pénicaud, Louis Casteilla, Anne Lorsignol

**Affiliations:** 1 RESTORE Research Center, INSERM, CNRS, EFS, ENVT, Université P. Sabatier, 31000 Toulouse, France; cecile.dromard-berthezene@inserm.fr (C.D.B.); geraldine.jourdan@envt.fr (G.J.); beatrice.cousin@inserm.fr (B.C.); Luc.penicaud@inserm.fr (L.P.); louis.casteilla@inserm.fr (L.C.); 2 Alan Edwards Center for Research on Pain, Department of Physiology and Cell Information Systems, McGill University, Montreal, QC H3A 0G1, Canada; lise.rabiller@mail.mcgill.ca

**Keywords:** opioids, opioid receptors, tissue repair, regeneration

## Abstract

Opioids are regarded as among the most effective analgesic drugs and their use for the management of pain is considered standard of care. Despite their systematic administration in the peri-operative period, their impact on tissue repair has been studied mainly in the context of scar healing and is only beginning to be documented in the context of true tissue regeneration. Indeed, in mammals, growing evidence shows that opioids direct tissue repair towards scar healing, with a loss of tissue function, instead of the regenerative process that allows for recovery of both the morphology and function of tissue. Here, we review recent studies that highlight how opioids may prevent a regenerative process by silencing nociceptive nerve activity and a powerful anti-inflammatory effect. These data open up new perspectives for inducing tissue regeneration and argue for opioid-restricted strategies for managing pain associated with tissue injury.

## 1. Tissue Repair: Scar Healing Versus Regeneration

After massive tissue injury, two opposite processes of tissue repair can occur: scar or regenerative healing. The former generally leads to an ongoing extracellular matrix deposition and fibrosis, always associated with failure in the recovery of the initial morphology of the damaged tissue and, at term, results in a decline of function [1]. In contrast, the regenerative healing allows for recovery of functional tissue that is morphologically similar to the original tissue. The regenerative capacity is heterogeneous between animal phyla and appears to decline through the evolutionary tree. In hydra and planaria, the entire organism can regenerate after section [2,3] and in non-mammalian vertebrates such as salamander or zebrafish, appendages and organs are able to regenerate [4,5,6,7,8]. Adult mammals mainly show scar healing, although some mammalian organs can really regenerate at embryonic and early postnatal stages [9,10,11,12,13,14]. These observations have led to the hypothesis that inhibitory elements locking the regenerative process develop early after birth. The identification of these blocking elements could provide attractive therapeutic targets for reactivating latent regenerative responses in adulthood.

Among the inhibitory elements, opioids are prime candidates. They alleviate the pain associated with injury through their release from the endogenous opioid system and their administration as effective analgesic agents. They are also able to modify the inflammatory response induced by injury. Although the role of opioids in tissue repair has been largely investigated in scar healing, it is not the case for true regenerative healing. Most of the studies have considered different repair processes (wound closure, leucocyte infiltration into the wound bed, angiogenesis and formation of the granulation tissue, re-epithelialization by stimulated keratinocytes, etc.) as an outcome of injury without a clear and precise distinction between scar healing versus regenerative healing and without considering the complete recovery of the initial architecture and function of the tissue. Moreover, neither positive nor negative effects have been confirmed by rigorously controlled studies in humans. Therefore, a definitive conclusion on the role of opioids in tissue repair, including in humans, is still under debate [15,16]. The first study on true regeneration was performed in 1973 in salamander and showed that opioid treatment after amputation inhibited hind limb regeneration while treatment with the opioid receptor antagonist naloxone accelerated the limb regeneration. This study thus suggests a deleterious effect of both medical treatments with opioids and endogenous opioids on regeneration [17]. Another study dealt with the regeneration of the pancreatic epithelium in induced pancreatitis in mice [18], showing that morphine prevented epithelium regeneration. In addition, in 2018, we demonstrated that opioid treatment inhibited both adult zebrafish caudal fin regeneration and subcutaneous adipose tissue regeneration in the adult MRL mouse [19], which displays uncommon regenerative capacities [19,20,21]. Moreover, regeneration could be induced in non-regenerative adult C57Bl6 mice by inhibiting opioid receptors with naloxone methiodide (NalM) [19]. This opioid receptor antagonist does not cross the blood–brain barrier and preferentially antagonizes mu opioid receptors (MORs) at the concentration used, which suggests an anti-regenerative effect of MOR activation outside of the central nervous system (CNS).

Taking into account that (1) rapid and necessary activation of both immune and sensory nervous systems systematically follows tissue damage and (2) endogenous opioids release or medical treatment with opioids after tissue injury can minimize activation of both systems, this review provides evidence that immunosuppressive and analgesic effects of opioids inhibit the regeneration process.

## 2. Opioids, Immune System, and Tissue Regeneration

### 2.1. Endogenous Opioids and Their Receptors

Endorphins, enkephalins, dynorphins, and nociceptins/orphanins represent the four families of endogenous opioids [22,23]. They are peptides of varying length and are mainly synthesized and released by well-identified neuronal sub-populations located in the CNS [23]. In addition to this neuronal production, opioids can also be synthesized and released by immune cells [24]. During the inflammatory response after tissue injury, neutrophils are the first source of endogenous opioids [25]. Subsequently, monocytes, macrophages, and then T and B lymphocytes may also secrete opioids [25]. These peripherally produced opioids have an analgesic effect by their binding to opioid receptors located on the peripheral terminals of sensory neurons, particularly those of nociceptive neurons that transduce noxious stimuli.

Four isoforms of opioid receptors have been identified. They are currently called MORs, delta opioid receptors, kappa opioid receptor, and opioid-receptor-like 1 receptors. Their endogenous ligands are β-endorphin, dynorphin, met-enkephalin, and nociceptin, respectively, although these peptides show significant cross-affinity for all opioid receptors [22,23,26,27]. All these receptors belong to the super family of 7-domain transmembrane receptors and are predominantly coupled to G proteins of the α_i_ or α_0_ type. Although several signaling pathways can be initiated by activation of opioid receptors and take part in the cellular effects of opioids, ligand binding to these receptors mainly leads to inhibiting adenylate cyclase, thus preventing cAMP production and protein kinase A activation [28,29].

### 2.2. Opioid Effects on the Immune System

Several studies, both in vitro and in vivo, report that opioids modulate immune responses mainly in the context of infection [30,31,32]. In 1979, Wybran et al., reported for the first time an immunosuppressive effect of opioids, showing that the administration of morphine inhibited the formation of “immune rosettes” (an association between human T lymphocytes and sheep red blood cells previously incubated with antigens), which reflects the inability of lymphocytes to produce antibodies [33]. This inhibition of “rosetting” was also observed with met-enkephalin and disappeared with naloxone administration. Since this initial study, the list of immunosuppressive effects of opioids has increased, showing direct and indirect effects on innate and adaptive immune cells. Concerning innate immunity, several studies demonstrated that opioids significantly decreased the ability of peritoneal macrophages to engulf and kill pathogens such as *Candida albicans* or *Streptococcus pneumonia* by the activation of MORs and delta opioid receptors [34,35,36]. This suppression of phagocytosis or microbicidal activity is mediated by the inhibition of reactive oxygen species (ROS) production [37,38] associated with a strong decrease in production of pro-inflammatory cytokines by macrophages [35]. In addition, opioids impair immune cell recruitment at the site of injury by (1) inhibiting leukocytes sticking to and rolling along blood vessels [39] and (2) disturbing chemotaxis by downregulating chemoattracting factors and cross-desensitization of chemokine receptors [30,31,40]. Thus, opioids dampen inflammation by their effects on both the number and activity of immune cells, an effect that is blocked by MOR antagonists or genetic deletion of the MOR [32].

### 2.3. Opioid Effects on Regeneration via Immune Cells

The immune system plays a major role in tissue regeneration after injury. Several studies have focused on the oxidative burst that characterizes the inflammation observed early after tissue injury. This early and transient production of ROS after injury is required for the regeneration process because pharmacological blockage of this production can inhibit tissue regeneration [6,19,41,42,43]. This observation was confirmed in mammals: treating non-regenerative C57Bl6 mice with NalM induced a rapid and significant increase in ROS production, essential for the NalM-induced adipose tissue regeneration, whereas control mice that received vehicle injection showed neither elevated ROS levels nor tissue regeneration. In addition, we demonstrated that this NalM-induced ROS production results from activating NADPH oxidase in neutrophils [19] (Rabiller et al., NPJ Regen Med, in press). Because NalM is a competitive antagonist of opioids, these results suggest that in control C57Bl6 mice, neutrophil ROS production is normally inhibited by endogenous opioids released at the injury site. Although the molecular mechanisms have not been investigated, opioids binding to MORs may directly modulate the addressing of NADPH oxidase at the plasma membrane [37]. This huge amount of ROS released by neutrophils likely allows for cleaning the injury site.

Because macrophages are also involved in the early steps of the immune response, their involvement in regeneration has been widely investigated. Their pharmacological depletion systematically led to regeneration failure in zebrafish, salamander, and the post-natal mouse, which demonstrates a pivotal role for macrophages in the regeneration process [14,44,45,46,47,48,49]. Using the adipose tissue regeneration model, we showed that NalM-induced tissue regeneration depends on efficient phagocytosis of apoptotic neutrophils by resident macrophages, thus leading to a fast resolution of inflammation (Rabiller et al., NPJ Regen Med, in press). As in other studies on different animal models, the precise mechanisms driving the shift from inflammatory macrophages to a “reparative” phenotype are still under investigation [47,50,51].

In conclusion, all these data suggest that (1) intense but time-controlled inflammation is required to guide tissue repair toward regeneration and (2) opioids, via their immunosuppressive effects, prevent the regeneration process.

## 3. Opioids, Nervous System, and Tissue Regeneration

### 3.1. Nervous System Organization and Tissue Injury

One of the fundamental aspects of organ or tissue regeneration is the ability of the body to integrate information about the location and intensity of the damage (“where and what is missing?”) to decide what exactly must be repaired. The nervous system, including the CNS and the peripheral nervous system (PNS), is optimally organized to ensure the body’s vital functions. In a constantly changing environment, the PNS allows for the perception of both internal and external information with the afferent pathways known as “sensory” pathways, allowing for feedback of information from the periphery to the CNS. Thereafter, the “motor” efferent pathways convey the adapted responses of the CNS to the target tissues in the periphery. The complementarity between the afferent and efferent PNS allows the organism to adapt to internal and external stimuli and function optimally under changing conditions.

### 3.2. Innervation and Regeneration

The importance of innervation in the regeneration process was first described in the salamander, in 1823 [4]. Tweedy John Todd reported that after surgical denervation of the sciatic nerve in the salamander, the hind limb failed to regenerate. After this key discovery, several studies have shown the requirement of the nervous system to ensure regeneration in the hydra, planaria, zebrafish, salamander, and the MRL mouse [21,52,53,54,55]. Most of these studies have indeed identified several neural cues able to control the formation of the blastema (where regenerative progenitor cells accumulate) and to mediate position signals [7,56,57].

Although the involvement of the nervous system in regeneration is not surprising, the precise identification of the nervous fibers required for regeneration remains poorly investigated. Two studies have addressed this issue in zebrafish [5,58]. Mahmoud et al. showed that, after partial amputation of the ventricle, cholinergic signaling is required for cardiomyocyte proliferation during heart regeneration. The authors obtained similar results in 1-day old neonatal mice after left vagotomy. These data suggest an involvement of the parasympathetic efferent fibers in heart regeneration, but this effect seems to be indirect via a downregulation of the inflammatory response [5]. One year later, Vriz et al., showed in zebrafish that surgical denervation of the caudal fin before its amputation led to loss of spontaneous regeneration [58]. Because the amputation site was exclusively innervated by sensory nerve fibers, the authors suggested that these fibers were the only ones required for caudal fin regeneration.

### 3.3. Opioids and Nociceptors

Among all types of sensory fibers, nociceptive neurons have drawn attention. Indeed, severe injury generates mechanical noxious stimuli that activate these specialized sensory neurons. In addition, the injured cells and the immune cells at the origin of the inflammatory response release multiple chemical mediators that can act on the peripheral nerve endings of the nociceptors. All these molecules constitute an acidic mixture what is commonly called the “inflammatory soup” and includes DAMPs (for damaged associated molecules pattern), ROS, histamine, serotonin, bradykinin, arachidonic acid derived lipid mediators such as prostaglandins or leukotrienes, cytokines, adenosine, ATP, protons, or growth factors such as NGF (nerve growth factor) [59]. Some of these products directly induce action potentials in nociceptive neurons, while others increase their sensitivity to harmful signals [60,61,62]. Therefore, the inflammatory response after tissue injury potentiates nociceptive neurons activation, increased neurotransmitter release in the spinal cord and activation of nociceptive ascending pathways, ultimately leading to pain perception. This unpleasant feeling or even “suffering” is normally circumscribed by endogenous opioids. Activation of opioid receptors in the spinal cord or the brainstem inhibits the transmission of nociceptive messages at a higher level [61,62]. Moreover, endogenous opioids released by immune cells during the inflammatory response exert their analgesic effect via the opioid receptors located on the peripheral endings of nociceptive neurons [63]. Indeed, inhibition of the cAMP/protein kinase A pathway after the binding of opioids to their receptors on nociceptive neurons endings leads to the closure of voltage-dependent calcium channels and/or the opening of G protein-regulated inward-rectifying potassium channels, inducing, in both cases, hyperpolarization of the nociceptive terminals [64]. This results in decreased or even cessation of action potential discharge. In addition, as primary afferent fibers, nociceptive neurons have both central and peripheral axons emanating from the cell body in the dorsal root ganglia, so that opioid inhibition of these neurons decreases the release of neurotransmitter at their central and peripheral endings [64,65,66,67,68].

### 3.4. Nociceptors and Regeneration

In this context, we can postulate that (1) nociceptive neurons are required for regeneration processes and (2) opioids are anti-regenerative molecules by inhibiting nociceptive neuron activity. According to the first part of the hypothesis, Wei et al., recently demonstrated in adult C57Bl6 mice that pharmacological activation of the transient receptor potential A1 (TRPA1) positive cation channels, expressed in certain nociceptive neurons [69,70,71], promotes regeneration in the ear hole closure model [69]. In general, after making a hole in the center of the outer ear pinna with a mechanical punch, the wound heals with a scar and remains open. In this study, topical application of imiquimod cream promoted complete closure of a 2 mm diameter punch, an effect that disappeared in TRPA1 knock-out mice [69]. Moreover, histological investigation revealed normal tissue architecture with hair follicles, sebaceous glands, and subcutaneous fat, without tissue fibrosis and scar formation [69]. The same results have also been obtained in a dorsal skin excision model. We confirmed the involvement of nociceptive nerves in the adipose tissue regeneration model using capsaicin treatment [72]. Indeed, this pharmacological depletion of TRPV1-positive nociceptive neurons, including TRPA1-positive neurons [70], prevented NalM-induced regeneration of adipose tissue in C57Bl6 mice (personal data). Finally, some nociceptive nerves release neuropeptides such as calcitonin gene-related peptide (CGRP) and/or substance P [61,62,70]. The peripheral release of these neuropeptides is responsible for the vasodilator and pro-inflammatory effects of nociceptive neurons [73,74]. Because the inflammatory response is required for regeneration (see first part of this review), activation of opioid receptors located on nociceptive nerve endings may contribute to an altered regeneration process by decreasing the peripheral release of these neuropeptides. Adipose tissue is innervated by CGRP- and substance P-positive fibers [75]. As CGRP is able to facilitate the migration of mesenchymal cells to a site of lesion [76] and as mesenchymal cells are adipocytes progenitors (i.e., mandatory cells for ontogeny and adipose tissue expansion) [77], the effect of this peptide on adipose tissue regeneration was investigated. Pretreatment of C57Bl6 mice with a selective CGRP receptor antagonist suppressed NalM-induced regeneration, whereas injection of CGRP alone (without NalM) was able to induce adipose tissue regeneration (personal data). These results suggest the involvement of CGRP signaling in the regenerative effect of opioid-receptor inhibition. Since (i) substance P is released after injury by the same subtype of nociceptive fibers as CGRP [70], (ii) this release is also inhibited by morphine [66], and (iii) substance P exerts similar effects as CGRP on local blood flow and immune cells attraction [78], substance P is likely able to drive the outcome of tissue repair to regeneration [79]. Nevertheless, to our knowledge, there are no studies showing the involvement of substance P in tissue regeneration even though its beneficial role in accelerating wound closure has been well demonstrated in the context of skin healing [80,81].

All these data support that (1) pain-sensing neurons must be activated to promote regeneration and (2) opioids prevent regeneration by silencing these neurons.

### 3.5. Nociception and Opioid System Development

Consistent data in the literature support a gradual decrease in regenerative potential during evolution and development associated with the acquisition of an increasingly complex immune system [8,82,83,84]. Therefore, the vestigial regenerative activity observed in fetal or young mammals would be due to a still immature immune system. The same hypothesis can be proposed with the nociceptive and opioid systems that undergo strong maturation at the postnatal stage. Although the last afferent sensory fibers to appear, nociceptive neurons are present and functional at birth. They express all the proteins involved in nociception, such as neurotransmitters, channels, or receptors. At this developmental stage, different inflammatory chemicals can sensitize them [85] and short as well as long hyperalgesia can be observed [86,87]. Nevertheless, at early postnatal ages, noxious stimulation often results in a prolonged electrical activity that lasts beyond the end of the stimulus. This effect decreases in amplitude and duration with age [88]. The thresholds for withdrawal from heat stimuli are also lower in younger animals, and sensitivity to formalin is 10-fold higher than in adults [86,89]. These exaggerated and sometimes inappropriate responses to noxious stimuli [90] disappear with the maturation of the nociceptive circuitry at the spinal and supra-spinal level that takes place between birth and weaning in rodents. The shaping of this complex sensory network results from decreased excitatory inputs in favor of more efficient inhibitory activity in the superficial laminae of the dorsal horn [91,92] in parallel with the progressive activation of the inhibitory descending pathways from the brainstem [93,94,95].

The opioid system also undergoes a postnatal maturation at the level of both endogenous peptides and their receptors. By postnatal day 21, the rat dorsal horn showed increased immunoreactivity to enkephalin [94], and several studies reported postnatal changes in subcellular location, density, and isoforms of opioid receptors expression in different brain areas [96,97,98,99]. Moreover, the coupling of MORs to G protein increases during postnatal development, which suggests that although these receptors are present at birth, their binding to opioids may not necessarily be associated with intracellular signaling activation [100]. This finding may account for the lower analgesic potency of morphine on noxious thermal stimulation in addition to the reorganization of the spinal connectivity that occurs over this time [101,102].

Collectively, these data suggest that the higher activity of the nociceptive network associated with an immature opioid system may be involved in the increased regenerative capacity of newborn and postnatal mammals.

## 4. What Is Known in Human-Being?

Although there is, to our knowledge, no clinical data available on the effects of opioids on regeneration per se, several reports have shown that these peptides are associated with impaired fracture healing and nonunion risk in human [103,104] as well as impaired wound healing [105]. Nevertheless, two studies report beneficial or neutral effects of opioids on painful skin lesions [106,107]. Therefore, the small number and the disparity of these studies (opioid subtype, concentration, application route, and time course of disease) make difficult to draw a conclusion [15,16,108].

Finally, the anti-regenerative effect of opioids described in this review is in line with the rethinking around the use of opioids during peri-operative period by anesthesiologists. In order to reduce, or even abolish, the well-known and deleterious side effects of opioids (respiratory depression, hyperalgesia, risk of dependence and chronic use, immunosuppression), opioid-sparing strategies, or strategies dealing with total suppression of opioids (also called opioid-free anesthesia) have emerged [109,110,111,112]. Opioid-sparing strategies consist in a non-opioid based multimodal pain management associating drugs with different mechanisms of action such as loco-regional techniques (peripheral nerve blocks, epidural analgesia), associated with paracetamol, NSAIDs (non-steroidal anti-inflammatory drugs), α2-agonists, ketamine, magnesium sulfate, intravenous lidocaine, etc., whose aim is to use the lowest effective opioid dose while providing satisfactory analgesia. This saving becomes total in the OFA (opioid free anesthesia) with a complete elimination of opioid usage. Drastically reducing the doses and adapting the analgesia to the real needs of each patient [109] should be beneficial to true tissue regeneration after the surgical act.

## 5. Conclusions

**Figure 1:**
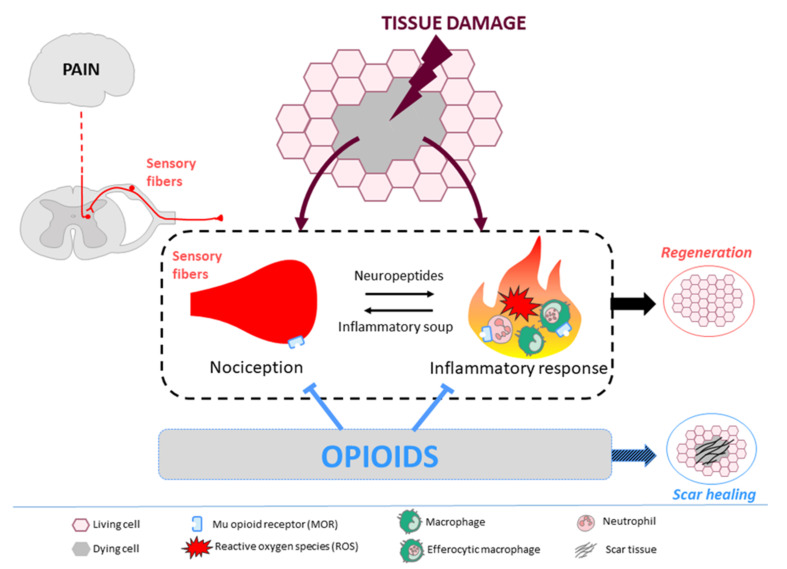
Schematic diagram illustrating the anti-regenerative effects of opioids during tissue repair.

After severe tissue injury, both the inflammatory response and nociceptive pathway activation collectively guide the outcome of tissue repair towards regeneration. However, endogenous opioids or opioids provided by post-operative medical treatment counteract the combined beneficial effects of inflammation and nociception on regeneration and instead promote scar healing. These opioid effects are mainly mediated by binding to MORs located on immune cells and nociceptive neurons, although the involvement of other opioid receptor subtypes cannot be excluded. Figure 1 provides a schematic overview of the opioid anti-regenerative effects. Although clinical studies questioning the role of opioids on regeneration are obviously lacking, opioids and their receptors could be strategic targets in regenerative medicine. The challenge is now to promote tissue regeneration rather than scar healing, while preventing the pain associated with tissue damage, with new analgesic treatments.

After tissue damage, both sensory nerve fibers and the immune system are activated. Inflammation occurs via the recruitment of reactive oxygen species-producing neutrophils, the activation of macrophages, and the expression/secretion of pro-inflammatory cytokines. Simultaneously, activation of sensory nerve fibers leads to (1) the generation of an ascending nociceptive message that is interpreted by the central nervous system as pain and (2) the peripheral release of neuropeptides (calcitonin gene-related peptide and/or substance P) that actively promotes the inflammatory response. Fine-tuning the amplitude and temporal pattern of inflammation controls the outcome of tissue repair. By silencing nociceptive neurons and preventing immune cell activation via binding to mu opioid receptors, opioids lead to an attenuated but sustained inflammation that does not allow for tissue regeneration.
